# Diagnostic and Therapeutic Dilemmas in Acute Stroke With Contrast Allergy: A Case Report

**DOI:** 10.7759/cureus.89458

**Published:** 2025-08-06

**Authors:** Kenneth Lee, Gemina Roncal, Elena Ioannou, Mohamed Abdulmajeed, Sakthivel Sethuraman

**Affiliations:** 1 General Internal Medicine, Luton and Dunstable University Hospital, Luton, GBR; 2 Stroke Medicine, Luton and Dunstable University Hospital, Luton, GBR

**Keywords:** contrast allergy, : ischemic stroke, normal brain imaging, stroke, neurology

## Abstract

Contrast allergy in acute stroke presents a significant challenge, as many aspects of management require the use of iodinated contrast agents. In patients with known contrast allergy, standard imaging techniques may be contraindicated. This case presents a woman in her 70s who developed right-sided facial droop, weakness, and slurred speech. Initial CT head imaging showed a likely hyperdense left middle cerebral artery, and she was thrombolyzed. However, CT angiography was not performed due to severe contrast allergy, limiting further mechanical thrombectomy if required. This case highlights the need for specialized management strategies for patients with contrast allergy in acute stroke and emphasizes the importance of timely decision-making in stroke management.

## Introduction

Stroke remains one of the leading causes of morbidity and mortality worldwide, with an estimated 12.2 million new strokes and 6.5 million stroke-related deaths reported annually, highlighting the critical need for rapid diagnosis and timely intervention to improve outcomes. Ischemic stroke is a medical emergency, defined as a clinical syndrome of presumed vascular origin characterized by rapidly developing signs of focal or global disturbance of cerebral function, lasting longer than 24 hours or leading to death. There are approximately 100,000 strokes annually in the UK, placing a significant burden on healthcare services [1]. Non-contrast CT is the initial imaging modality of choice in acute stroke to exclude hemorrhage; however, advanced imaging with contrast-enhanced CT angiography (CTA) or CT perfusion is often required to guide reperfusion therapies. These imaging modalities require iodinated contrast medium, which complicates both diagnosis and treatment in patients with a history of severe contrast allergy [2].

Although contrast reactions are generally uncommon, they can delay appropriate diagnosis and subsequent treatment of large vessel occlusions (LVOs), which account for approximately 25% of all ischemic strokes [3]. Severe contrast allergy is rare. In a study of 90,473 patients over 15 years at a single center, only 0.43% of iodinated contrast medium injections resulted in adverse reactions. Of these, 19 were severe (0.02%), including two deaths, while most were mild or self-limiting [4]. Nevertheless, clinicians managing patients with stroke symptoms and a history of contrast allergy must carefully balance the risks and benefits of administering contrast. Furthermore, documentation of previous contrast reactions is often imprecise in electronic health records, further complicating decision-making in emergency situations [5].

At present, there are no UK-wide guidelines addressing the management of acute stroke in patients with contrast allergy, resulting in variability in practice and potential delays in care [6]. The time-sensitive nature of acute stroke exacerbates this clinical dilemma, with every minute of delay in reperfusion therapy resulting in the loss of approximately 1.9 million neurons [7]. International experience has demonstrated that emergent premedication protocols can be safely implemented, but these approaches have not been systematically evaluated or widely adopted in UK practice [8]. Other imaging techniques that do not require iodinated contrast have also been tested, with varying success [9].

## Case presentation

A woman in her 70s was pre-alerted to the emergency department of a district general hospital after being found on the floor by her daughter with right-sided facial droop, right-sided weakness, and slurred speech. On examination by the stroke team, her National Institutes of Health Stroke Scale (NIHSS) score was six for right-sided drift, right-sided facial droop, and hemianopia, raising clinical suspicion of a left middle cerebral artery (MCA) stroke. She was last seen well at 08:10 AM and arrived at the emergency department at 09:05 AM, approximately 55 minutes from symptom onset. Non-contrast CT (NCCT) was performed at 09:25 AM, corresponding to 75 minutes from onset.

**Figure 1:**
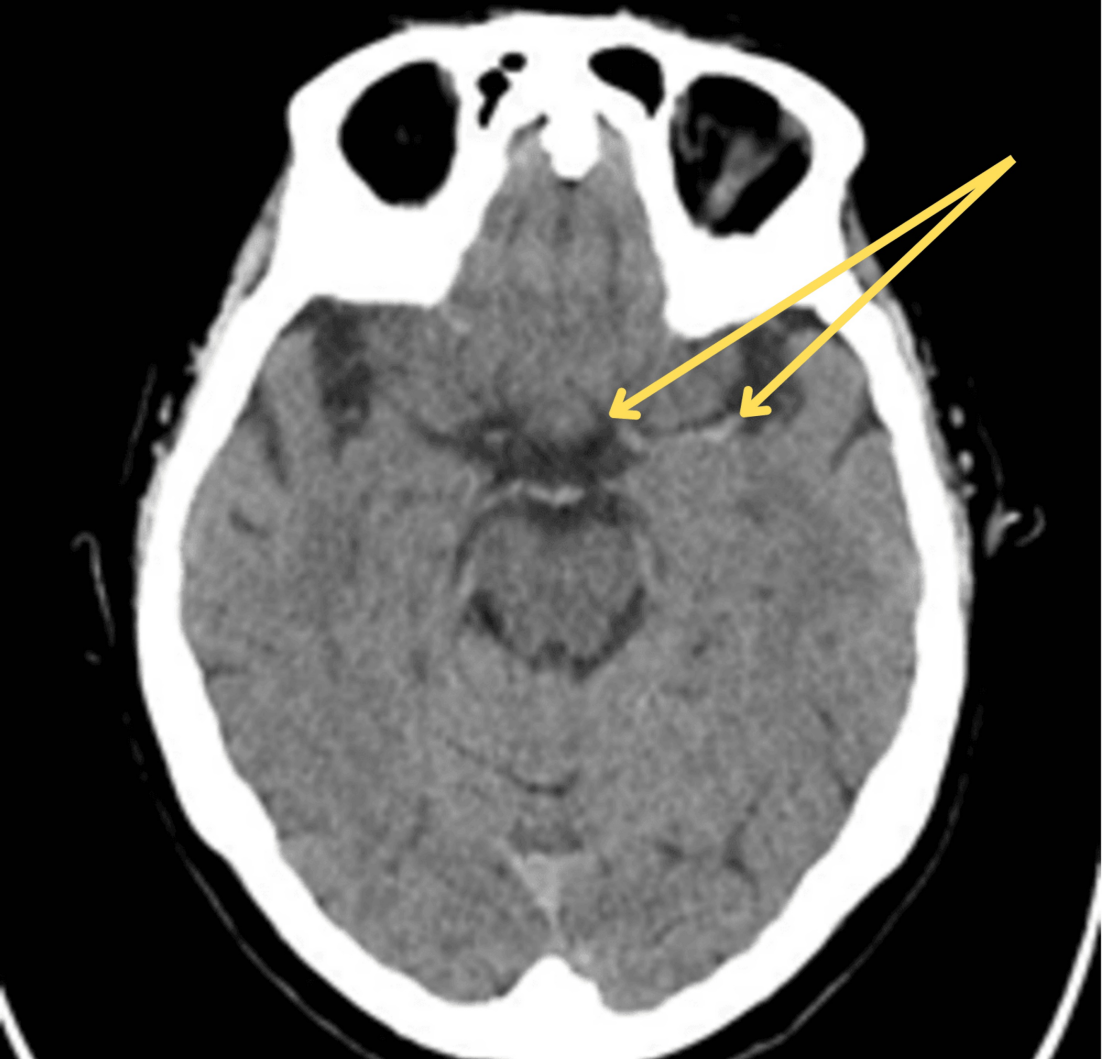
Axial section of non-contrast CT head showing hyperdense left middle cerebral artery

Her medical history included transient ischemic attack, myocardial infarction, previous liver and colon cancer resulting in a right hemicolectomy, previous breast cancer with mastectomy, and hypertension. She had a documented severe anaphylactic reaction to contrast medium. Her initial plain CT head showed a hyperdense left MCA, likely representing thrombosis (Figure 1). The stroke team considered further CTA to guide thrombectomy, given the high suspicion of LVO. A non-ionic, low-osmolar iodinated contrast agent (iohexol) was intended, as it is the current standard due to its significantly lower risk of hypersensitivity reactions compared to older ionic, high-osmolar agents. This was discussed with the patient and her family, but they declined due to the patient’s prior severe anaphylaxis.

**Figure 2:**
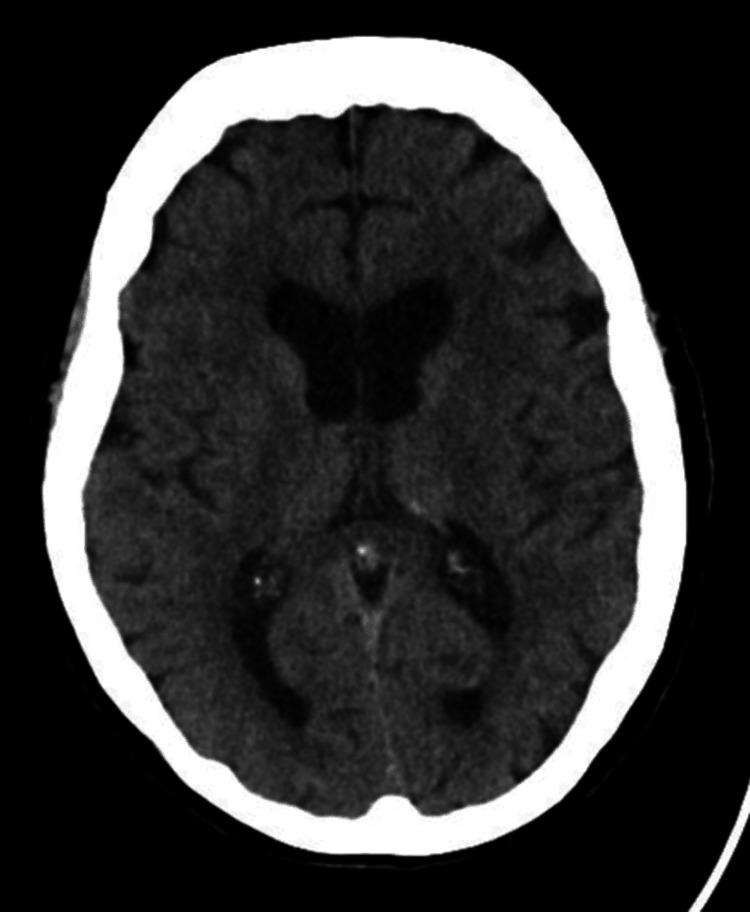
CT head showing no acute parenchymal infarction

The patient was reviewed by the on-call stroke consultant and deemed suitable for thrombolysis. Prior to starting thrombolysis, labetalol was administered to treat raised blood pressure. Her blood pressure improved following labetalol, and a bolus of alteplase was given. However, her slurred speech worsened after the bolus, prompting a repeat plain CT head, which showed no acute parenchymal infarction (Figure 2). The alteplase maintenance infusion was started but subsequently paused as her systolic blood pressure rose above 180 mmHg despite multiple intravenous labetalol boluses. Eventually, the alteplase infusion was withheld, and a labetalol infusion was initiated.

**Figure 3:**
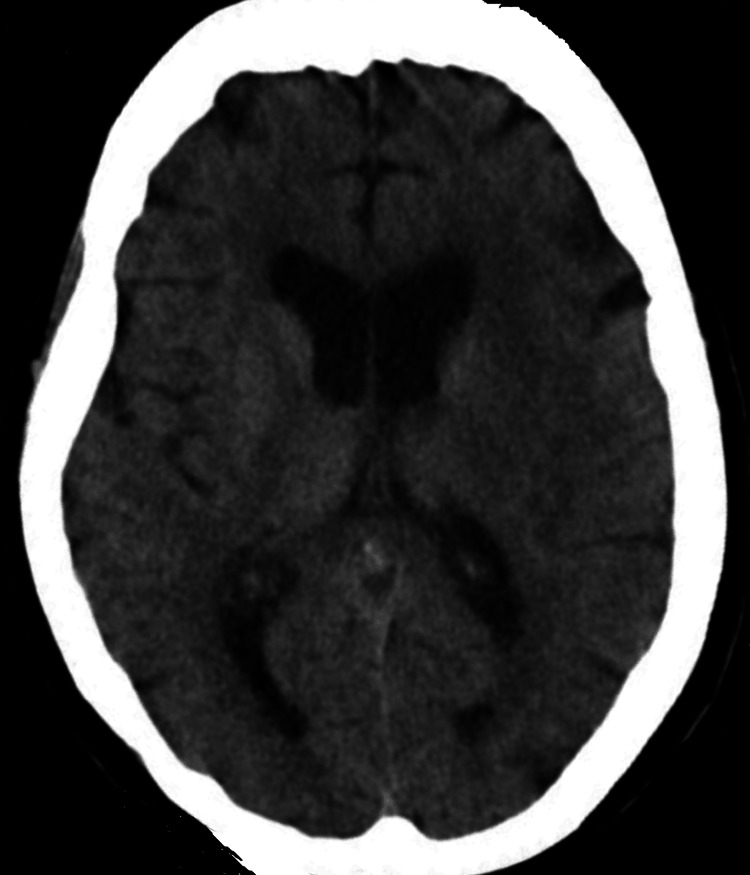
Repeat CT head showed no haemorrhage and no evidence of established infarct

**Figure 4:**
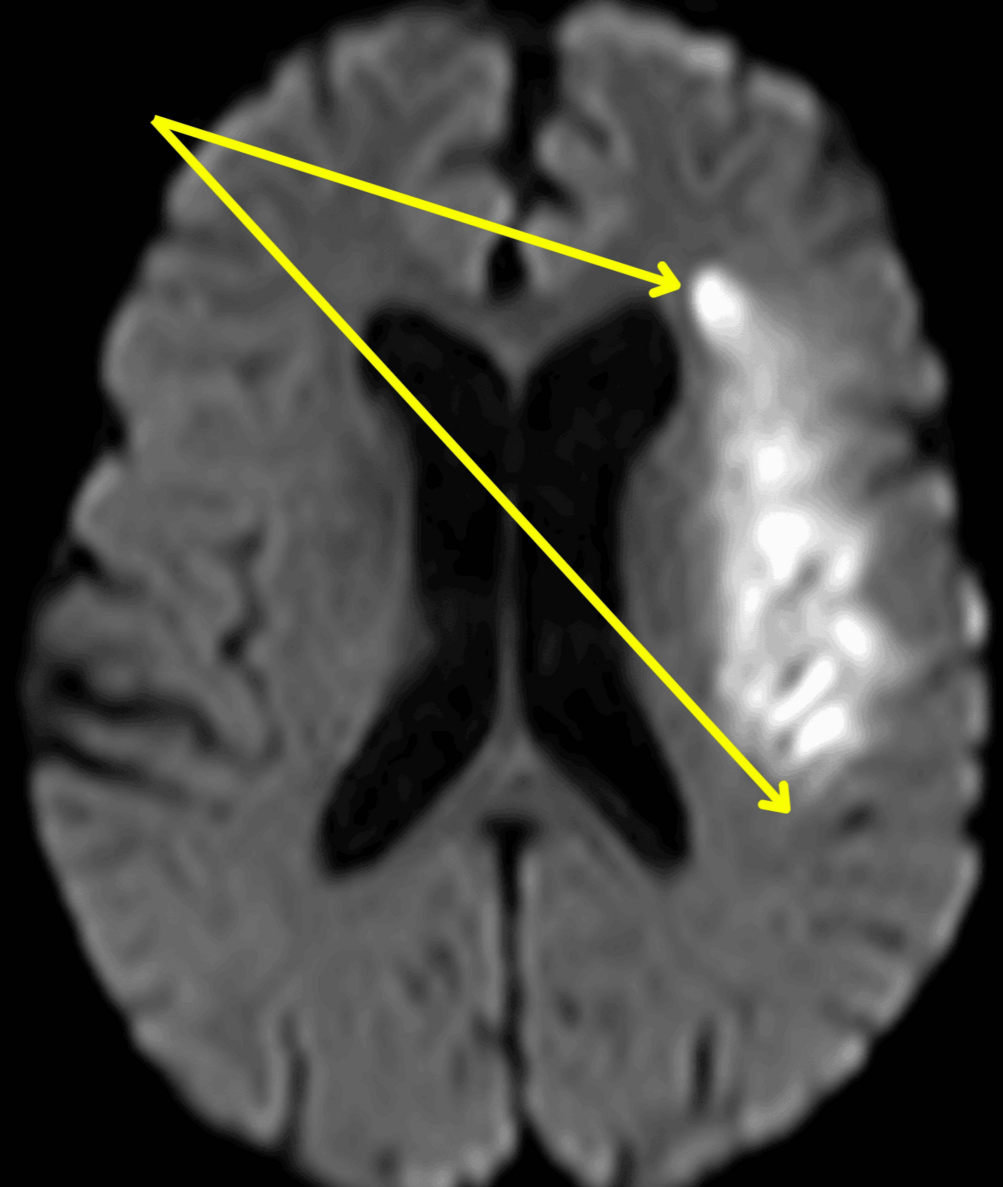
Axial section of MRI head showing acute non-hemorrhagic infarct in the left middle cerebral artery (MCA) territory Axial section of MRI head showing an acute non-haemorrhagic infarct in the left MCA territory. The infarction is indicated by the arrow.

A repeat CT head the following day showed no hemorrhage and no evidence of established infarct (Figure 3), and she was started on 300 mg of aspirin. An MRI head performed two days later showed an acute non-hemorrhagic infarct in the left MCA territory (Figure 4).

She remained hospitalized for additional medical reasons and was assessed by speech and language therapists, physiotherapists, and occupational therapists. She was discharged home with two weeks of 300 mg of aspirin.

At follow-up eight weeks after discharge, the patient had been transferred to a rehabilitation unit, having become fully dependent on activities of daily living. She continued with appropriate secondary prevention measures.

## Discussion

The management of contrast allergies in acute stroke patients represents a significant clinical challenge. Contrast-enhanced imaging, particularly CTA and CT perfusion, has become integral to acute stroke pathways, enabling the identification of LVOs and assessment of salvageable brain tissue. However, in patients with known contrast allergy, as in this case, clinicians must carefully balance diagnostic needs against the risk of anaphylaxis.

This case aligns with existing guidance regarding the use of non-contrast CT in initial stroke evaluation. According to the National Institute for Health and Care Excellence (NICE), non-contrast CT remains the first-line modality to exclude hemorrhage in suspected stroke [1]. The decision to proceed with thrombolysis after non-contrast imaging and blood pressure optimization follows standard practice. The patient’s contrast allergy precluded further vascular imaging, reflecting the caution advised by Cochran et al., who highlighted the risks associated with iodinated contrast use [2].

Although the patient was a candidate for mechanical thrombectomy based on clinical findings, the lack of CTA due to her allergy limited further intervention. Tonetti et al. emphasized the importance of minimizing contrast exposure in allergic patients, even in emergent stroke settings [3]. While this conservative approach avoided immediate risk, it also limited the opportunity to escalate therapy with thrombectomy.

Premedication protocols with corticosteroids and antihistamines have been proposed to reduce the risk of severe contrast reactions and allow necessary imaging. Studies by Virador et al. and Fusco et al. showed that premedication can facilitate the safe administration of contrast in select patients [4,5]. In this case, no premedication was attempted due to time constraints and the patient’s history of severe anaphylaxis, highlighting the variability in clinical practice and institutional preparedness.

In this case, the clinical team engaged the patient’s family in a discussion about the risk-benefit balance between irreversible neurological injury and the potential for a treatable, yet life-threatening, allergic reaction. Although rapid premedication with diphenhydramine and methylprednisolone was theoretically an option, it was not pursued due to the acute time-sensitive nature of the situation and the limited effectiveness of steroid premedication when given immediately prior to contrast exposure. Time-of-flight magnetic resonance angiography (MRA) was also considered; however, it was not available under our emergency stroke protocol, and the delay required for MRI acquisition would have compromised timely thrombolytic decision-making. These real-world limitations highlight the challenges clinicians face when managing acute stroke in patients with contrast allergies.

Alternative imaging techniques are gaining attention. Fusco et al. suggested that time-of-flight MRA (TOF-MRA) may serve as a non-contrast option in evaluating LVOs, though its sensitivity is lower compared to contrast-enhanced studies [5]. Boujan et al. demonstrated that contrast-enhanced MRA provides superior diagnostic clarity compared to TOF-MRA, but its use is limited in patients with contrast allergies [6]. While rare cases of hypersensitivity to both iodinated and gadolinium-based contrast agents have been reported, current evidence indicates there is no clinically significant cross-reactivity between these two contrast classes due to their distinct chemical structures [7].

Gadolinium-based contrast has been successfully used in select cases. Male et al. described its use in acute stroke thrombectomy when iodinated contrast was contraindicated [8]. Similarly, De Albóniga-Chindurza et al. reported intra-arterial gadolinium use as an alternative for vascular imaging in patients with severe allergies [9]. Despite these findings, our case did not explore gadolinium due to concerns regarding nephrotoxicity and time-sensitive decision-making in the acute setting.

Another important factor is the absence of standardized national protocols for stroke patients with contrast allergies. While local NHS guidelines exist, such as those from Worcestershire Acute Hospitals NHS Trust and NHS Lanarkshire [10,11], these are not uniformly applied across the UK. The National Optimal Stroke Imaging Pathway (NOSIP) provides comprehensive imaging guidance but lacks specific protocols for contrast-allergic populations [12]. This variability highlights the need for unified national guidance to ensure consistent and equitable stroke care for this vulnerable group.

## Conclusions

Contrast allergy in acute stroke presents significant diagnostic and therapeutic challenges. There is an urgent need for national guidelines and research to standardize care pathways and ensure equitable access to advanced stroke therapies for patients with contrast allergy, particularly regarding vascular imaging and eligibility for reperfusion therapies. Alternative management strategies include premedication protocols with steroids and antihistamines, non-contrast MRI techniques, and the use of gadolinium-based contrast instead of iodinated contrast. Multidisciplinary collaboration and local protocol development are essential to optimize outcomes in this patient population. National guidelines are needed to standardize care and ensure equity in access to advanced stroke therapies for patients with contrast allergy.
